# Toward a new focus in antibiotic and drug discovery from the *Streptomyces arsenal*

**DOI:** 10.3389/fmicb.2015.00461

**Published:** 2015-05-13

**Authors:** Sergio Antoraz, Ramón I. Santamaría, Margarita Díaz, David Sanz, Héctor Rodríguez

**Affiliations:** Departamento de Microbiología y Genética, Instituto de Biología Funcional y Genómica, Consejo Superior de Investigaciones Científicas, Universidad de SalamancaSalamanca, Spain

**Keywords:** antibiotics, *Streptomyces*, co-culture, interactions, signals

## Abstract

Emergence of antibiotic resistant pathogens is changing the way scientists look for new antibiotic compounds. This race against the increased prevalence of multi-resistant strains makes it necessary to expedite the search for new compounds with antibiotic activity and to increase the production of the known. Here, we review a variety of new scientific approaches aiming to enhance antibiotic production in *Streptomyces*. These include: (i) elucidation of the signals that trigger the antibiotic biosynthetic pathways to improve culture media, (ii) bacterial hormone studies aiming to reproduce intra and interspecific communications resulting in antibiotic burst, (iii) co-cultures to mimic competition-collaboration scenarios in nature, and (iv) the very recent *in situ* search for antibiotics that might be applied in *Streptomyces* natural habitats. These new research strategies combined with new analytical and molecular techniques should accelerate the discovery process when the urgency for new compounds is higher than ever.

## Introduction

Since pioneering work leading to the isolation of the antibiotic streptomycin in 1943 by Waksman et al. (Jones et al., 1944), huge progress has been made in the elucidation of the molecular basis and mechanism behind the production of these biological weapons by the *Streptomyces* genus. Following this initial discovery, thousands of compounds produced by these microorganisms have been described and utilized in order to fight infections, and they comprise over two-thirds of all known antibiotic compounds (Omura, 1992; Berdy, 2005; Hopwood, 2007). Nowadays, widespread antibiotic resistance (McArthur et al., 2013; Mak et al., 2014; Lin et al., 2015) has rendered a large number of these compounds ineffective and is currently urging the scientific community to push the boundaries of classical microbiology toward a faster and more efficient secondary metabolite search.

Antibiotic biosynthesis is carried out by a high number of proteins encoded by genomic clusters and is tightly regulated (Bibb and Hesketh, 2009). Normally, there is specific regulation for each product, mediated by *Cluster-Situated Regulators* and also global or pleiotropic mechanisms of regulation that can control several pathways at the same time (Rokem et al., 2007; Martín and Liras, 2012). Therefore, there are complex regulatory networks that control the onset of production of the secondary metabolites (Liu et al., 2013). These networks respond to multiple signals, many of which are still unknown, and therefore empirical methods are needed to trigger the production of cryptic secondary metabolites.

**Figure 1:**
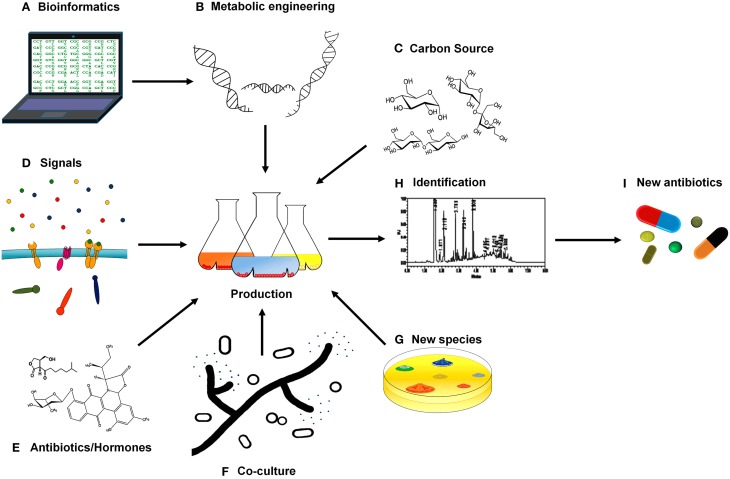
**Schematic overview of new approaches for antibiotic discovery in** ***Streptomyces*****. (A,B)** Biosynthetic clusters and regulatory elements can be predicted with bioinformatic tools, opening up new possibilities to metabolic engineering. **(C)** Modification of the culture medium that is crucial in antibiotic production such as different carbon sources. **(D)** Searching for the signals that activate different regulatory systems triggering antibiotic production. **(E)** Communication molecules like antibiotics and hormones may also boost secondary metabolism. **(F)** Co-culture of *Streptomyces* species with fungi and bacteria might simulate interspecies interactions and thus induce antibiotic production. **(G)** The discovery of new *Streptomyces* species could also reveal new compounds with antibiotic activities. **(H)** The compounds produced have to be identified and isolated. **(I)** All these approaches contribute to the elucidation of the nature and activity of new antibiotics.

Genome sequencing combined with *in silico* prediction has revealed that microorganisms of the genus *Streptomyces* harbor a high number of secondary metabolism clusters (Aigle et al., 2014; Bachmann et al., 2014; Ikeda et al., 2014). Bioinformatics and “omics” based engineering has become a powerful tool in this field, allowing the identification of secondary metabolite gene clusters and their possible products by similarity searching (Chaudhary et al., 2013) (Figure 1A). The use of techniques developed in recent years in metabolic engineering will also be of tremendous value and the perfect complement in this urgent quest for new antibiotic compounds (Aigle and Corre, 2012; Weber et al., 2015). Changing promoters, introducing biosynthetic clusters in others species, or rewiring transcriptional and post-transcriptional regulation are methods for unveiling new antibiotics or modifying products previously discarded to obtain new molecules with antibiotic activity (Figure 1B). Removal of endogenous secondary metabolites gene-clusters, based on the previously described competition for precursors, has been also shown as an alternative for improving antibiotic production (Komatsu et al., 2010; Gómez-Escribano and Bibb, 2011, 2014). Although all these techniques have shown their efficacy, when cultured in axenic conditions most bacteria express a limited number of these clusters. That is the reason that makes the unlocking of *Streptomyces* cryptic pathways (potentially abundant) one of the most feasible methods for antibiotic(s) discovery in this counter clock race against multi-resistant strains. Moreover, new approaches designed with a view to awake silent or cryptic pathways will also surely result in the discovery of highly valuable secondary metabolites with antifungal, herbicidal, anti-cancer, immunosuppressive, anti-inflammatory, antihelmintic, or antiviral activities, amongst others, widening the interest for the research in this area.

## Studying bacterial sensors to find key molecules triggering antibiotic burst: the nutritional signals

It is widely known that media composition has a great impact on microbial secondary metabolites, comprising of activators of signaling cascades that trigger their production (Yang et al., 2010). Based on systematic culture modification of easily accessible parameters, some strategies such as “One Strain-Many Compounds” (OSMAC) were proven successful more than a decade ago, leading to the isolation of up to 20 different metabolites from species of *Streptomyces* (Bode et al., 2002). In particular, chemical compounds present in *Streptomyces* niches but not found in culture media are thought to play a role in cryptic metabolite activation as signals in sensory mechanisms, triggering regulatory cascades responsible for the tuning of the secondary metabolites synthesis. That is the case of N-acetylglucosamine, the monomer of chitin that has been shown to act as a signal, mediated by the DasR global regulator (Rigali et al., 2008), that controls antibiotic production (Swiatek et al., 2012; Nazari et al., 2013). The recently reported cellobiose-induced production of thaxtomin A by *Streptomyces scabies* is also of interest (Francis et al., 2015). More generally, the effect of carbon source(s) in antibiotic production is also a subject of study (Figure 1C), since some of the most used carbon sources, in which bacteria are growing more “comfortably,” repress secondary metabolism (Sánchez et al., 2010). We could possibly use less “efficient” carbon sources, which however might induce more antibiotic production in an effort to establish a balance between growth yield and antibiotic yield.

Regarding signal translation into responses, two component systems (TCS) are the main signaling pathways in *Streptomyces* and their role in the antibiotic production complex regulatory network has just been started to be decoded (Rodríguez et al., 2013). Nevertheless, the signals that activate the different *Streptomyces'* TCS remain mostly unknown and just a few of them have been described to date (Figure 1D). As an example, in *S. coelicolor* phosphate is the signal triggering PhoP/R (Sola-Landa et al., 2003), nitrogen balance seems to be related with AfsQ (Shu et al., 2009) and DraR/K (Yu et al., 2012) regulation, the level of iron seems to be the signal activating AbrA1/2 activator (Rico et al., 2014) and the presence of heme-oxidative stress provokes SenS/R reaction (Bogel et al., 2007; Ortiz de Orue Lucana and Groves, 2009). Most of the signals sensed by the TCS present in the genome of the *Streptomyces* spp. sequenced so far (more than 100 genomes) remain elusive. Phosphate, nitrogen and iron are compounds present in laboratory culture media. However, other compounds found in nature but absent in lab culture might also act as the signals triggering other TCSs responses (or different regulatory mechanisms) and therefore controlling antibiotic production. So, the addition of low concentration of rare earth elements to the culture medium may activate the secondary metabolism in *S. coelicolor* and in other *Streptomyces* species. Scandium has been the one studied more in depth but yttrium, lanthanum, cerium, and europium can also provoke antibiotic production boost (Kawai et al., 2007). The molecular mechanism under this induction, however, has not been described yet.

New developments in *Streptomyces* research, also linked with nutrients, since their depletion is coupled with sporulation, are exploring new solutions in order to wake silent pathways through morphological differentiation, namely sporulation recovery. As has been recently described, physiological differentiation is tightly linked to secondary metabolism and therefore recovering sporulation capacities of some *Streptomyces* might also lead to the discovery of new compounds (Chater, 2013; Kalan et al., 2013). Another nutrient-related deficiency of axenic cultures is the absence of siderophores. Some *Streptomyces* species are defective in the production of these iron-chelating compounds and need to utilize those released by other species in order to differentiate, produce secondary metabolites or even grow in lab conditions (Yamanaka et al., 2005; Eto et al., 2013; Lambert et al., 2014). Therefore, the addition of purified siderophores or the co-culture of non-producer species with siderophore producers are strategies also related with nutrient supply that might be used to awake silent pathways.

## Spying microbial conversations: bacterial hormones and antibiotics as signals

The presence of antibiotics is an important piece of information for the microorganism in order to respond to threats (as a signal of the competitors presence) or even to coordinate efforts with other antibiotic producing neighbors (combining strategies in order to repel a common menace). Therefore, the addition of certain antibiotics or bacterial hormones in low concentrations to the culture medium might also be an alternative for antibiotic production stimulation (Figure 1E). Over the last 5 years there have been several reports on the role of antibiotic compounds as auto-inducers of antibiotic production (Romero et al., 2011). Even more, the importance of molecules previously described as antimicrobials, in inter-specific communication between *Streptomyces* species and, as a consequence, in the regulation of antibiotic production has been recently described (Nodwell, 2014). For example, the antibiotic jadomycin B, an angucycline, produced by *Streptomyces venezuelae*, triggers different antibiotic production levels in *S. coelicolor* depending on the concentration (Wang et al., 2014a). Additionally, hormones also play a role in communication between bacteria. Among them, gamma-butyrolactones have been demonstrated to promote antibiotic production in many streptomycetes (Sidda and Corre, 2012). These molecules are involved in cell to cell communication processes (quorum sensing) in which bacteria use the production and detection of autoinducers in order to synchronize gene expression and population growth (Garg et al., 2014). For example, A-factor, a gamma-butyrolactone, autoinduces morphological differentiation and secondary metabolite production in *S. griseus* (Horinouchi and Beppu, 2007). Recently, an exogenous butyrolactone has also been showed to increase validamicyn antibiotic production in *Streptomyces hygroscopicus* 5008 (Tan et al., 2013). Although, many *Streptomyces* species are apparently capable of synthesizing gamma-butyrolactones (Takano, 2006) a recent research has also made clear that antibiotic production can also be triggered by different hormones like avenolide in *Streptomyces avermitilis* or methylenomycin furans in *S. coelicolor* (Corre et al., 2008; Kitani et al., 2011). Regulation of antibiotic production by microbial signaling molecules such as hormones and foreign antibiotics is widespread. A better understanding of the nature and functions of these signals could drive us to their potential use as activators of silent pathways.

## Exploiting microbial communal living: co-cultures

Routine laboratory work with *Streptomyces*, as with other microorganisms, has been basically done in axenic cultures. However, antibiotic functions can only be understood in the context of *Streptomyces*' habitat. Traditionally, antibiotics have been considered biological weapons that allow the bacteria to compete with others microorganisms, either by killing them or inhibiting their growth. Nevertheless, antibiotics are also signals that trigger adaptive responses (Yim et al., 2007; Fajardo and Martínez, 2008; Aminov, 2009). Antibiotics have evolved as a result of interactions (mainly competitive but also cooperative) with other organisms and their natural role has just started to be elucidated (Davies, 2009, 2013). It is, therefore, not unreasonable to think that the presence of either foreign neighboring species in its environmental niches or its buddy's signals could trigger different patterns of secondary metabolites production (Vetsigian et al., 2011).

Although streptomycetes have been considered as normal inhabitants of soil, recent studies show that *Streptomyces* species are also frequent in different habitats in the underwater world, mainly in sediments from shallow and deep water habitats and marine dwelling animals, and as symbionts of plants and invertebrates (Seipke et al., 2012; Raveh et al., 2013). Besides, the variety of organisms that share these different niches with *Streptomyces* is huge. In order to achieve a laboratory scenario that resembles more closely the environmental conditions, co-culture of two or three species has emerged as a powerful tool (Figure 1F). This aims to mimic real simple situations in nature that will facilitate the discovery of new secondary metabolites. Although just a limited number of experiments have been carried out to date using this new approach, results are promising. One of the seminal experiments in this area used different combinations of streptomycetes in co-culture to produce the stimulation of antibiotic production and differentiation (Ueda et al., 2000). More recently, co-cultures of several *Streptomyces* species with different fungi and bacteria have reported an induction of new molecules or the stimulation of previously known compounds either in *Streptomyces*, in the other partner or even in both partners (Seyedsayamdost et al., 2012; Watrous et al., 2013; Moody, 2014). An example shows that pairwise co-culture of *Streptomyces coelicolor* with five different actinomycetes produces a range of compounds of unknown identity, among them, at least 12 different desferrioxamines, that were not produced when *S. coelicolor* was grown under pure culture (Traxler et al., 2013). Similarly the co-culture of the predator bacteria *Myxococcus xanthus* with *S. coelicolor* showed that *S. coelicolor* increases actinorhodin production in order to repel the invader when it senses the presence of the predator (Pérez et al., 2011). In some cases substance-mediated induction has been discarded, with cell-to-cell interaction being the causative agent of antibiotic biosynthetic pathways “decryption.” Thus, the interaction of *Streptomyces* with mycolic acid-containing bacteria such as *Tsukumurella pulmonis* in co-cultures provokes the synthesis of new natural antibiotic products (i.e., alchivemycin A by *S. endus*) although not mediated via any chemical substance (Onaka et al., 2011). Presence of plant pathogens has also been shown to trigger the production of secondary metabolites able to suppress *Verticillium dahlia*, such as prodiginines, by *S. lividans* (Meschke et al., 2012). *Streptomyces* products obtained in the presence of plant invaders are becoming an interesting tool for biocontrol initiatives that are being developed in order to fight plant plagues (Taechowisan et al., 2005; de Oliveira et al., 2010; Meschke and Schrempf, 2010; Cuesta et al., 2012; Meschke et al., 2012; Palaniyandi et al., 2013a,b). One step further of “natural co-culture” lies in the culture of *Streptomyces* in the presence of human pathogens pushing the evolutionary mechanisms of *Streptomyces* toward the biosynthesis of natural compounds able to outcompete the pathogen. So, *Streptomyces clavuligerus* co-cultured with methicillin resistant *Staphylococcus aureus* was able to synthesize holomycin, a *S. aureus* chemical inhibitor not detected in axenic cultures (Charusanti et al., 2012). Other interesting interactions are shown in Table 1. In this way, co-culture allows the induction of secondary metabolism even when signals that trigger the response remain unknown or the induction is due to a combination of factors that is hardly reproducible in axenic conditions.

**Table 1:** ***Streptomyces*** **co-cultures involved in antibiotic production**.

**Co-cultured species**	**Effects**	**References**
*Streptomyces coelicolor*–five actinomycetes	Production of multiple cryptic compounds and antibiotics (i.e., prodiginines and actinorhodines)	Traxler et al., 2013
Combinations of 76 *Streptomyces* spp.	Stimulation of various antibiotics	Ueda et al., 2000
*Streptomyces coelicolor*–*Bacillus subtilis*	Increase of undecylprodigiosin production. Earlier onset of production	Luti and Mavituna, 2011
*Streptomyces* spp.*–Tsukamurella pulmonis*	Production of novel antibiotics (i.e., alchivemycin A by *S. endus*)	Onaka et al., 2011
*Streptomyces coelicolor–Myxococcus xanthus*	Increase of actinorhodin production in *S. coelicolor*	Pérez et al., 2011
*Streptomyces clavuligerus–Staphylococcus aureus*	Production of holomycin	Charusanti et al., 2012
*Streptomyces cinnabarinus*–*Alteromonas* sp.	Induction of lobocompactol production	Cho and Kim, 2012
*Streptomyces* sp. Mg1–*Bacillus subtilis*	Production of chalcomycin A	Barger et al., 2012
*Streptomyces* sp.–Proteobacteria	Production of the antibiotic resistomycin	Carlson et al., 2015
*Streptomyces coelicolor*–*Corallococcus coralloides*	Increase of antibiotic production of undecylprodigiosin and earlier onset	Schäberle et al., 2014
*Streptomyces fradiae* 007–*Penicillium* sp. WC-29-5	Production of four aromatic polyketides	Wang et al., 2014b
*Streptomyces lividans–Bacillus subtilis*	Induction of prodiginine production	Vargas-Bautista et al., 2014
*Streptomyces lividans–Verticillium dahliae*	Increase of antibiotic production of prodiginines	Meschke et al., 2012

## Taking the lab to the field: *in situ* culture for antibiotic discovery

Uncultured bacteria make up approximately 99% of all species. These “undomesticated” microorganisms are potentially a huge unexplored source of antibiotic compounds (Lewis, 2013). The recent publication by Ling et al. of new methods for *in situ* cultivation of previously uncultivable microbial species opens up a new world of possibilities enabling the search for natural products in previously inaccessible sources (Ling et al., 2015). These new methods are, respectively, based on cultivation of the microorganism in their natural environment using a multichannel device that allows diffusion of nutrients and growth factors (Nichols et al., 2010) and on the use of siderophores as growth factors, to microorganisms out of their environment (D'onofrio et al., 2010). As a proof of concept of the *in situ* culture approach, teixobactin, a new antibiotic produced by the Gram-negative bacteria *Elephteria terrae* with excellent activity against Gram-positive pathogens, was discovered in an extract obtained using iChip devices (Ling et al., 2015). Many new species are being described every day and it is thought that most of *Streptomyces* species remain undiscovered to date, foreseeing unlimited possibilities for future antimicrobial discovery (Figure 1G).

## Identifying compounds: technical advances in secondary metabolite detection

Although production of cryptic secondary metabolites is the main goal, it is important to consider other aspects, such as the identification of the compounds produced in each condition. This is the first step for the purification and elucidation of their structures and activity. Previous technical problems have been solved by emerging analytical techniques like nanospray desorption electrospray ionization (NanoDESI) and matrix-assisted laser desorption ionization–time of flight (MALDI–TOF) imaging mass spectrometry that allow researchers to gain an *in situ* global chemical view of bacterial secretions (Watrous et al., 2013; Fang and Dorrestein, 2014; Hsu and Dorrestein, 2015). Therefore, the use of new advanced techniques applied in addition to classical detection/identification methods such as High Performance Liquid Chromatography (HPLC) or Mass spectrophotometry (MS) (Figure 1H) will also be crucial in this crusade against resistant pathogens.

## Concluding remarks

A new universe of possibilities for antibiotic discovery (Figure 1I) is opening up through multiple strategies whereby the aforementioned genetic techniques may be complemented by deeper studies on bacterial relationships and elucidation of the compounds serving as signals for regulator systems. As a consequence of both, these novel techniques and the need for new and more effective products, the following years might therefore present a new golden age for antibiotic discoveries after 70 years of the pioneering discoveries in *Streptomyces*.

### Conflict of interest statement

The authors declare that the research was conducted in the absence of any commercial or financial relationships that could be construed as a potential conflict of interest.
